# Comparative performance of bagging and boosting ensemble models for predicting lumpy skin disease with multiclass-imbalanced data

**DOI:** 10.1038/s41598-025-23846-7

**Published:** 2025-11-10

**Authors:** Hagar F. Gouda, Fatma D. M. Abdallah

**Affiliations:** Animal Wealth Development Department (Biostatistics subdivision), Faculty of Veterinary Medicine, Zagazig University, Zagazig, 44511 Sharkia Egypt

**Keywords:** Bagging, Ensemble learning, Lumpy skin disease, Vaccination, Multi class imbalance, SMOTE, Biological techniques, Computational biology and bioinformatics, Zoology, Diseases, Risk factors, Mathematics and computing

## Abstract

Ensemble machine learning (ML) algorithms, such as bagging and boosting, are powerful decision-support tools that enhance disease prediction and risk management in the veterinary field. Lumpy Skin Disease (LSD) poses a significant threat to livestock health and results in substantial economic losses. This study aims to predict LSD using 1,041 data records collected from six Egyptian governorates between June 2020 and October 2022. The dataset exhibits a multiclass imbalance with three outcome classes: Dead (6%), Diseased (32%), and Healthy (62%). To address this imbalance, we applied SMOTE, Random Oversampling (ROS), and Random Undersampling (RUS). Five ensemble models: Decision Tree (DT), Random Forest (RF), AdaBoost, Gradient Boosting (GBoost), and XGBoost were evaluated on both imbalanced and balanced datasets, with hyperparameter tuning via grid search and 10-fold cross-validation. Our findings highlight the superior performance of the RF model combined with ROS (RF-ROS), achieving the highest accuracy (82%) and AUC (0.93), followed by balanced XGBoost (81.25%, AUC = 0.93). AdaBoost and GBoost also improved significantly after oversampling and tuning. SHAP analysis identified vaccination status as the most important predictor, emphasizing targeted interventions. These results demonstrate that combining resampling with hyperparameter tuning enhances ML performance on imbalanced veterinary data.

## Introduction

Lumpy skin disease virus (LSDV) poses a serious threat to cattle production, causing both acute and subacute illness in cattle and water buffalo. All breeds are susceptible, with lactating cows and calves being at higher risk^1^. Monitoring risk factors such as deworming methods, vaccination, grazing patterns, use of disinfectants, and fly repellents characteristics can aid in determining their impact on LSD risk^2^. Additionally, factors including breed, age, season, water supply, feeding methods, importation of breeding stock, and exposure to other species such as birds and insects play important roles in the prevalence of LSD^3^. From an economic standpoint, LSD represents an existential threat to cattle-dependent economies, notably in Asia and Africa. This disease reduces dairy productivity, and in outbreak resulted in significant losses due to abortions, weight loss, and reduced infertility. The World Organization for Animal Health has classified LSD as a notifiable disease, requiring timely reporting^4^. In many countries, vaccination is the primary means of controlling and preventing LSD^5,6^. However, effective preventive measures are still limited. Restricting the movement of sick cattle, implementing quarantine, and sacrificing infected animals are strongly advised^7^.

Early and precise detection is critical for effective epidemic management and mitigation. This can be achieved by integrating advances in computer vision and artificial intelligence^8^. The modelling of LSD risk contributes significantly to addressing challenges in LSD epidemiology and control, particularly in the areas of risk factors, disease transmission, diagnosis and forecasting, and intervention techniques^9^. Machine learning (ML) techniques like Artificial Neural Networks (ANN), Decision Trees (DTs), and Random Forest (RF) can considerably improve the accuracy of LSD prediction based on geographical and climate features. This powerful tool could help to build targeted monitoring and awareness initiatives, as well as preventive measures like vaccination campaigns, in areas prone to LSDV infection^10^. However, class imbalance, which occurs when the majority class outnumbers the minority class by a large amount, poses a significant barrier to ML prediction accuracy^11^. This issue is particularly prevalent in veterinary medicine, where rare outcomes such as mortality are significantly underrepresented, further impairing the model’s ability to accurately learn and predict these minority classes^12^. The impact of class imbalance is more severe in multi-class classification compared to binary classification. As a result, there has been growing attention on the challenges of multi-class imbalance classification in recent years^13^. So, building powerful ML algorithms with high model accuracy requires careful attention to class imbalance, which affects data quality^14^. A widely adopted strategy to address class imbalance is resampling, which aims to balance the dataset either by reducing the majority class (undersampling) or expanding the minority class (oversampling). Undersampling removes instances from the majority class, thereby improving computational efficiency but potentially compromising the loss of valuable information and introducing bias, especially in complex datasets^15^. The common methods include random undersampling and Tomek Links, which eliminate overlapping majority samples. In contrast, oversampling increases minority class representation by duplicating existing instances, preserving data integrity but risking overfitting^15,16^. To mitigate this, SMOTE (Synthetic Minority Over-sampling Technique) was introduced by Chawla^17^, which generates synthetic samples to enhance generalization. Choosing the appropriate resampling method depends on multiple factors, including the dataset’s structure, size, and characteristics, as well as research objectives. As highlighted in^18^, resampling effectiveness is influenced not just by imbalance ratios, but also by the intrinsic nature of the data, emphasizing the need for context-specific strategies. Ensemble learning is considered one of the most effective strategies for addressing class imbalance in machine learning tasks^19^. By aggregating predictions from multiple models, hence reducing forecasting errors and improving accuracy^20^. Consequently, it has been regarded as one of the most effective ML methods^21^. Bagging and boosting are two powerful ensemble learning methods that improve prediction accuracy by combining multiple models. Bagging, or bootstrap aggregation, involves training several models on randomly selected subsets of the training data and aggregating their outputs through majority voting or averaging. In contrast, boosting trains models sequentially, with each model focusing on correcting the errors of its predecessor by assigning greater weight to misclassified instances. By leveraging the strengths of multiple learners, both bagging and boosting enhance predictive performance and model robustness^22^.

In the veterinary field, ensemble ML models have emerged as powerful tools for improving predictive accuracy and robustness, particularly in veterinary epidemiology, where complex and imbalanced datasets are common. Table 1 summarizes recent studies that have applied ensemble techniques for predicting livestock disease, highlighting the models used, methods for handling data imbalance, and key findings of those studies. However, a clear research gap remains: most studies have focused on binary classification problems and have not systematically evaluated ensemble models in multiclass imbalance scenarios, especially for LSD. To the best of our knowledge, no prior study has comprehensively compared the performance of various ensemble models, including Decision Tree (DT), Random Forest (RF), Adaptive Boosting (AdaBoost), Gradient Boosting (GBoost), and eXtreme Gradient Boosting (XGBoost), specifically for multiclass predictions of LSD. Moreover, the impact of widely used resampling techniques such as the Synthetic Minority Over-sampling Technique (SMOTE), Random Oversampling (ROS), and Random Undersampling (RUS), within these ensemble frameworks, has not been fully explored. This study aims to fill this gap by conducting a thorough comparative analysis of these ensemble models, combined with different resampling strategies, on a real-world multiclass imbalanced LSD dataset. Specifically, it seeks to answer the following research questions:

**Table 1:** Recent applications of ensemble machine learning models in veterinary disease prediction with a focus on class imbalance Handling.

Ensemble ML Models	Class Imbalance Handling	Reference	Disease & Task	Key Contributions
RF, DT, MLP	SMOTE	^23^	Surgery & survival prediction in livestock	RF showed highest performance (Surgery: 85.83%, AUC: 0.906), SMOTE enhanced predictions.
AdaBoostM1, LogitBoost, Vote, Bagging	Not addressed	^24^	Brucellosis risk in cattle	Ensemble ML can be useful tools for predicting the risk of brucellosis infection in cattle and identifying the most important associated risk factors.
RF, Bagging, Boosting, DT, Rotation Forest.	SMOTE, Undersampling	^25^	Abortion prediction in dairy herds	- Balanced datasets improved accuracy - under sampling out performed SMOTE
RF, Classification Tree, CHAID	Not treated	^26^	FMD outbreak prediction	- ML models showed accuracy from acceptable to excellent without resampling

1. Are there significant differences in predictive performance between bagging and boosting algorithms?
2. Does addressing data imbalance improve predictive performance, and which resampling technique is most effective?
3. Can hyperparameter tuning enhance model performance even when data remains imbalanced?

**The key Contributions of this study are summarized as follows**:

- This study provides a comprehensive comparative evaluation of five ensemble learning algorithms (DT, RF, AdaBoost, GBoost, and XGBoost) for the prediction of LSD on a real-world, multiclass-imbalanced dataset.
- This study systematically investigates the impact of three distinct resampling techniques (SMOTE, ROS, and RUS) on the performance of these models in addressing class imbalance for LSD prediction.
- This study identifies the RF algorithm combined with ROS (RF-ROS) as the most effective approach for predicting LSD under the studied conditions, particularly for the critical minority “Dead” class.
- This study offers insights into the effectiveness of hyperparameter tuning in improving the performance of ensemble models on both imbalanced and resampled LSD datasets.
- The study emphasizes the importance of translating ML results into interpretable insights for practical use in real-world veterinary settings. The application of SHAP analysis proved effective, revealing that vaccination status is the most significant predictor of LSD risk.

## Materials and methods

### Source of the dataset

This study included data from a total of 1041 cows across 6 governorates, collected between June 2020 and October 2022. The animals were sourced as follows:

- **Field Outbreaks**: Cattle from 31 herds were included if the herds experienced suspected lumpy skin disease (LSD) outbreaks during the study period. Herds were identified through notifications from local veterinary authorities and active surveillance programs. All animals within these herds underwent a clinical examination.
- **Veterinary Clinic Admissions**: An additional 275 cases were included from cattle admitted to the Zagazig University Veterinary Clinic in Sharkia governorate, Egypt. These admissions were either referrals from field veterinarians or direct presentations by owners for suspected LSD.

## Sampling approach

A census sampling approach was used. In each affected herd and clinic admission group, all available animals were examined and included based on clinical presentation and laboratory confirmation. No random or systematic sampling was applied.

## Inclusion criteria

- Cattle were included if they belonged to herds with at least one animal showing clinical signs consistent with LSD (e.g., skin nodules, fever, lymphadenopathy) during the outbreak period.
- For clinic cases, only animals presenting with clinical suspicion of LSD were considered.
- Both field and clinic cases were further classified based on clinical outcome at the time of data collection:
  - **Dead**: Animals that died as a direct result of LSD, confirmed by clinical history and, where possible, post-mortem findings.
  - **Diseased**: Animals showing clinical signs of LSD but surviving at the time of data collection.
  - **Healthy**: Animals from the same herds or clinic admissions that showed no clinical signs of LSD during the outbreak period and tested negative by PCR.

## Case confirmation

All suspected LSD cases (both dead and diseased) were confirmed by a combination of clinical diagnosis and laboratory testing. Skin nodule biopsies and nasal swabs were collected and tested for LSDV DNA using PCR at the Virology Department of the Animal Health Research Institute, Dokki, Giza, following established protocols as described in a previous study^27^.

## Ethical compliance and consent

All methods were conducted in accordance with the relevant guidelines and regulations, including those of the Zagazig University Animal Care and Use Committee (Permit No. ZU-IACUC/2/F/114/2022) and the ARRIVE guidelines. All procedures involving animals were explained to and approved by the cattle owners, and informed consent was obtained prior to data collection.

### Feature engineering and data preprocessing

Data on demographic and management variables (breed, sex, age, season, feeding/watering system, introduction of new cattle, vaccination status) were collected for each animal using standardized questionnaires and farm records. The data were accessed through a data sharing-agreement with the study author. Both the laboratory analytical output and the necessary questionnaire response data were recorded, coded, and filtered in Microsoft Excel before being uploaded to R. We utilized the R programming language along with the following packages for data processing and model development: tidyverse^28^, readxl^29^, RandomForest^30^, caret^31^, xgboost^32^, adabag^33^, and gbm^34^.

The clinical cases (categorized as Healthy, Diseased, or Dead) were used as the target multiclass variable. We used both univariable and multivariable multinomial logistic regression to identify the key factors influencing lumpy skin disease (LSD). Variables with a *P-vaue* < 0.05 were considered statistically significant and retained as important predictors. The data revealed severe class imbalance as the ratios are (Dead 0.06, Diseased 0.32 and Healthy 0.62). The predictor features are presented in Table 2. The data are categorical, so we preprocess the data by one-hot-encoding. OneHotEncoder is used to convert the categorical features into binary form and subsequently intoa sparse [0,1] matrix, which was then fed into the model. The dataset was then split into two subsets: 80% for training and 20% for testing the model’s predictive performance. To address the class imbalance in the training data, we applied three resampling techniques: Synthetic Minority Over-sampling Technique (SMOTE), Random Over-sampling (ROS), and Random Under-sampling (RUS).

**Table 2:** Distribution of LSD clinical outcomes across predictor Categories.

Predictors	Category	Healthy	Diseased	Dead
Breed	Holstein	392	201	28
	Balady	190	85	15
	Mixed	65	47	18
Sex	Male	298	89	22
	Female	349	244	39
Age	0_to_1y	112	98	24
	1_to_3y	418	195	31
	more_than3y	117	40	6
Season	Winter	510	128	19
	Summer	72	145	32
	Autumn	54	48	8
	Spring	11	12	2
Feeding System	CommunalSystem	582	285	53
	Separate	65	48	8
Introduction of New Cattle	Yes	127	198	42
	No	520	135	19
Vaccine Type	Neethling	401	112	12
	Unvaccinated	98	158	39
	SheepPox	82	45	7
	Unknown	66	18	3

## Hyperparameter tuning procedure

To tune the classification algorithms, a customized grid search was used, and the sets of hyperparameter values were evaluated using 10-fold cross-validation (10-fold CV) repeated 5 times. The range of the hyperparameter values and their justification are presented in Table 3. After obtaining the optimal hyperparameter values, each classification model was trained and tested, and the accuracy, precision, recall, F1 score and ROC-AUC were extracted.

**Table 3:** Selected hyperparameter values and tuning justification based on repeated 10-Fold Cross-Validation.

Models	Hyperparameters	Custom range of values	The best tuned Values	Justification
DT	**Cp** (Complexity Parameter)	0.001, 0.01, 0.05	0.01	This value achieved the lowest cross-validation error and helps prevent overfitting, which Decision Trees are prone to when trained on imbalanced data.
	**Minsplit** (Minimum Split Size)	5 to 20	20	A relatively higher value (20) was selected to ensure that nodes are not split too early, which helps prevent the tree from modeling noise in the data, a critical consideration when dealing with minority classes in imbalanced datasets.
	**Maxdepth** (Maximum Tree Depth)	3 to 30	15	Limiting the maximum depth of the tree to 15 strikes a balance between allowing the tree to capture complex patterns and preventing it from becoming too specialized to the training data.
	**minbucket**	3 to 10	5	A value of 5 was chosen to balance complexity and generalization by avoiding small terminal nodes that may capture noise, while preserving key data distinctions.
RF	**Mtry** (Number of Variables randomly Sampled at Each Split)	2 to 12	12	This value improved performance by evaluating more features at each split, capturing key interactions and reducing variance—reflected in higher F1-scores and recall for minority classes.
	**Ntree** (Number of Trees)	50 to 3000	100	Using 100 trees strikes a balance between computational efficiency and achieving stable performance.
XGBoost	**Nrounds** (Number of Boosting Iterations)	100, 1000	1000	This value was chosen to allow the XGBoost model sufficient iterations to learn the data patterns without overfitting.
	**Max_depth** (Maximum Tree Depth)	3, 5, 7	6	A depth of 6 was found optimal, deep enough to capture non-linear patterns but shallow enough to avoid overfitting and balance bias-variance tradeoff in ensemble boosting.
	**Eta**	0.01, 0.05, 0.1, 0.3	0.3	A moderate learning rate of 0.3 ensured rapid yet stable convergence. Combined with 1000 boosting rounds, it allowed the model to learn efficiently without overfitting.
	**Gamma**	0, 0.1, 0.5, 1	0	Setting gamma to 0 allows the algorithm to make any split that improves loss, which could be beneficial in capturing complex relationships in the data.
	**Colsample_bytree** (Feature Sampling Ratio per Tree)	0.5, 0.7, 1	1	Using all columns for each tree helps to provide each tree with a complete view of the data, which can lead to better individual tree performance.
	**Min_child_weight** (Minimum Sum of Instance Weight in a Child)	1, 5, 7	1	Lower values make the model more sensitive to class imbalance. A value of 1 allowed the model to capture minority class patterns effectively, which is crucial in our imbalanced dataset.
	**Subsample** (Row Sampling per Tree)	0.5, 0.7, 1	1	Using the full training instance helps prevent underfitting and capture maximum information. A value of 1 was selected as it provided the best accuracy and F1-score. Lower values slightly reduced performance, likely due to information loss in small samples.
	**Booster** (Boosting Method)		**gb-tree**	Tree-based boosting (gbtree) was selected as it is best suited for tabular, structured data and is known for strong performance in classification tasks, particularly under class imbalance.
Adaboost	**Mfinal**	10, 50, 100, 200	10	mfinal is the number of iterations (i.e., weak learners). A small value like 10 was chosen to prevent overfitting and reduce computational load while still boosting performance.
	**Maxdepth**	1, 3, 5, 10	10	Controls the depth of the decision trees used as base learners. Depth 10 allows capturing more complex patterns while avoiding overly deep trees that might overfit.
	**Coeflearn**	Breiman, Freund, Zhu	Breiman	Specifying the Breiman weighting method adjusts the influence of each classifier in the ensemble based on its error rate. Moreover “Breiman” was selected as it yielded better balance between sensitivity and specificity in this study.
GBoost	**N.tree**	50, 100, 200, 300, 400, 500	300	Setting the number of trees to 300 allows for a complex model capable of capturing intricate relationships in the data, while still being computationally tractable.
	**Interaction.depth**	1, 2, 3, 5	3	Controls the depth of each tree (i.e., the level of interaction captured). A depth of 3 offers a good balance between model complexity and generalization.
	**Shrinkage**	0.01, 0.1, 0.2, 0.3	0.3	It allows the model to learn relatively quickly while still maintaining stability and avoiding drastic changes in the ensemble’s predictions.
	**N.minobsinnode**	5, 10, 15, 20	5	Sets the minimum number of observations required in the terminal nodes. A small value like 5 helps the model be more sensitive to minority class patterns.

These final values were selected because they offered the best trade-off between accuracy and generalization, particularly for underrepresented classes. The model with these settings demonstrated improved F1-score and balanced accuracy across folds, suggesting effective learning without overfitting.

## Ensemble learning algorithms

Five ML algorithms, including DT, RF, AdaBoost, GBoost, and XGBoost, were trained to predict the clinical case of lumpy skin disease. Their performance was evaluated using metrics derived from the confusion matrix to determine the best model. Each model was assessed both with default parameters and after hyperparameter tuning, and evaluated before and after balancing the training set.

### Decision tree

Classification and Regression Tree (CART) is a non-parametric tree-structured recursive partitioning method that hierarchically organizes the most influential variables to predict a response. This method works by recursively partitioning the data based on predictor-response relationships, forming a tree-like structure of decision rules. The root node initiates the process, followed by internal nodes representing further splits, and leaf nodes representing final classifications. The algorithm iteratively seeks optimal splits to maximize predictive accuracy^35^.

In our study, we applied the DT to predict a multiclass LSD status response variable $\:{Y}_{\left(\mathrm{l}\mathrm{u}\mathrm{m}\mathrm{p}\mathrm{y}\:\mathrm{s}\mathrm{k}\mathrm{i}\mathrm{n}\:\mathrm{d}\mathrm{i}\mathrm{s}\mathrm{e}\mathrm{a}\mathrm{s}\mathrm{e}\:\mathrm{c}\mathrm{a}\mathrm{s}\mathrm{e}\right)}$ on the basis of *p* risk predictors: $\:{X}_{\left(\mathrm{a}\mathrm{g}\mathrm{e}\right)}$, $\:{X}_{\left(\mathrm{s}\mathrm{e}\mathrm{x}\right)}$, $\:{X}_{\left(\mathrm{s}\mathrm{a}\mathrm{e}\mathrm{s}\mathrm{o}\mathrm{n}\right)}$, $\:{X}_{\left(\mathrm{b}\mathrm{r}\mathrm{e}\mathrm{e}\mathrm{d}\right)},\:{X}_{\left(\mathrm{v}\mathrm{a}\mathrm{c}\mathrm{c}\mathrm{i}\mathrm{n}\mathrm{a}\mathrm{t}\mathrm{i}\mathrm{o}\mathrm{n}\:\mathrm{s}\mathrm{t}\mathrm{a}\mathrm{t}\mathrm{u}\mathrm{s}\right)},$ $\:{X}_{\left(\mathrm{g}\mathrm{r}\mathrm{a}\mathrm{z}\mathrm{i}\mathrm{n}\mathrm{g}\:\mathrm{s}\mathrm{y}\mathrm{s}\mathrm{t}\mathrm{e}\mathrm{m}\right)}$, $\:{X}_{\left(\mathrm{i}\mathrm{n}\mathrm{t}\mathrm{r}\mathrm{o}\mathrm{d}\mathrm{u}\mathrm{c}\mathrm{t}\mathrm{i}\mathrm{o}\mathrm{n}\:\mathrm{o}\mathrm{f}\:\mathrm{n}\mathrm{e}\mathrm{w}\:\mathrm{c}\mathrm{a}\mathrm{t}\mathrm{t}\mathrm{l}\mathrm{e}\mathrm{s}\right)}$ observed on a learning sample of *N* units.

While growing, the CART algorithm performs binary recursive partitioning of the *N* data instances into increasingly homogeneous subsets (nodes). At each internal node *t*, all possible splits $s \in S$ across the covariates are evaluated, and the best split is chosen to maximize the reduction in impurity:

$$\:\varDelta\:\boldsymbol{I}\left(\boldsymbol{s},\boldsymbol{t}\right)=\boldsymbol{i}\left(\boldsymbol{t}\right)-\boldsymbol{P}\left({\boldsymbol{t}}_{\boldsymbol{L}}\right).\:\boldsymbol{i}\left({\boldsymbol{t}}_{\boldsymbol{L}}\right)-\boldsymbol{P}\left({\boldsymbol{t}}_{\boldsymbol{R}}\right).\:\boldsymbol{i}\left({\boldsymbol{t}}_{\boldsymbol{R}}\right)$$

Where:

*i(t)* impurity measure at node t, and *t*_*L*_, *t*_*R*_ are the resulting left and right child nodes, and *P(t*_*L*_*)*, *P(t*_*R*_*)* are the proportions of observations falling into *t*_*L*_ and *t*_*R*_, respectively.

The CART algorithm uses the Gini impurity index to select the best split variable. For a dataset *D* with *m* categories, the impurity is measured by the Gini index as:

$$\:\boldsymbol{G}\boldsymbol{i}\boldsymbol{n}\boldsymbol{i}\:\left(\boldsymbol{D}\right)=\:1-\sum\:_{\boldsymbol{i}=1}^{\boldsymbol{m}}{\left({\boldsymbol{P}}_{\boldsymbol{i}}\right)}^{2}$$

with *P*_*i*_ is the probability recording in *D* belongs to class *C*_*i*_ and is estimated by $\:\frac{|\mathrm{C}\mathrm{i},\mathrm{D}|}{\left|\mathrm{D}\right|}$^36^. The sum is computed over *m* classes.

The recursive partitioning process continues until no further meaningful splits can be made. To avoid overfitting, the fully grown tree is pruned using a cost-complexity criterion *(CP)*:

$$\:{\boldsymbol{C}}_{\boldsymbol{\alpha\:}}\left(\boldsymbol{T}\right)=\boldsymbol{C}\left(\boldsymbol{T}\right)+\boldsymbol{\alpha\:}.\:\left|\stackrel{\sim}{\boldsymbol{T}}\right|$$

Where *C(T)* represents the overall misclassification error of the tree, aggregated from the individual misclassification errors *c(t)* at each node, and $\:\left|\stackrel{\sim}{\boldsymbol{T}}\right|$ is the number of terminal nodes, and α ≥ 0 is a penalty parameter controlling tree complexity. This pruning helps select the most predictive and generalizable subtree, often based on cross-validation performance^36^.

### Random forest

RF is widely recognized as one of the most effective ensemble methods, largely due to its simplicity and high predictive performance^37^. It employs bootstrap aggregation (bagging) to combine multiple decision trees, enhancing the overall predictive performance. The feature with the lowest Gini index is selected as the optimal feature for data splitting:

$$\:\boldsymbol{G}\boldsymbol{i}\boldsymbol{n}\boldsymbol{i}\:\boldsymbol{i}\boldsymbol{n}\boldsymbol{d}\boldsymbol{e}\boldsymbol{x}\:\left(\boldsymbol{x}\right)=\:1-\sum\:_{\boldsymbol{i}=1}^{\boldsymbol{n}}{\left({\boldsymbol{x}}_{\boldsymbol{i}}\right)}^{2}$$

Notably, RF is an excellent predictive model for handling missing data, efficiently manages imbalanced datasets to reduce mistakes, and aids in determining the importance of variables in categorization. The algorithm uses a voting mechanism among the sub-algorithms to determine performance. The algorithm’s strength stems from the collective voting performance of similar trees within the forest. Meanwhile, RF is ideal for high-dimensional datasets with many features. It reduces variance by averaging and utilizing deep decision trees created from several subsets of training data. While this strategy may introduce some bias and reduce interpretability, it often results in a significant improvement in model performance^38^. Despite being accurate, RF is often considered a black-box model due to its limited interpretability, as the ensemble of deep trees makes it difficult to isolate individual variable effects. In this study, RF was implemented using the “randomForest” package (version 4.6–12). In RF, each base learner (i.e., decision tree) has access to a random subset of feature vectors^39^, which is defined as follows:

$$\:\boldsymbol{x}=\left({\boldsymbol{x}}_{1},{\boldsymbol{x}}_{2},\dots\:,{\boldsymbol{x}}_{\boldsymbol{p}}\right),$$

where ***p*** is the dimensionality of the available vector for the base learner. The main goal is to find the prediction function as *f*(*x*) that predicts the *Y* parameter. The prediction function is defined as follows:

$$\:\boldsymbol{L}\left(\boldsymbol{Y},\:\boldsymbol{f}\left(\boldsymbol{x}\right)\right),$$

Here, *L* is known as the loss function, and the goal is to minimize the expected value of the loss. For classification applications, zero-one loss is a common choice. The function is defined as follows:

$$\:\boldsymbol{L}\left(\boldsymbol{Y},\boldsymbol{f}\left(\boldsymbol{x}\right)\right)=\boldsymbol{I}\left(\boldsymbol{Y}\ne\:\boldsymbol{f}\left(\boldsymbol{x}\right)\right)=\left\{\begin{array}{c}0,\:if\:Y=\left(\boldsymbol{x}\right),\\\:1,\:otherwise\end{array}\right.$$

To create an ensemble, a set of base learners comes together. The base learners are defined as follows:

$$\:{\boldsymbol{h}}_{1}\left(\boldsymbol{x}\right),\:{\boldsymbol{h}}_{2\:}\left(\boldsymbol{x}\right),\dots\:,\:{\boldsymbol{h}}_{\boldsymbol{j}}\left(\boldsymbol{x}\right),$$

For classification applications, the voting will be based on the following equation:

$$\:\boldsymbol{f}\left(\boldsymbol{x}\right)=\boldsymbol{a}\boldsymbol{r}\boldsymbol{g}\boldsymbol{m}\boldsymbol{a}\boldsymbol{x}\sum\:_{\boldsymbol{j}=1}^{\boldsymbol{J}}\boldsymbol{I}(\boldsymbol{y}={\boldsymbol{h}}_{\boldsymbol{j}}(\boldsymbol{x}\left)\right)$$

The fundamental RF algorithm steps are summarized as:

**Figure:**
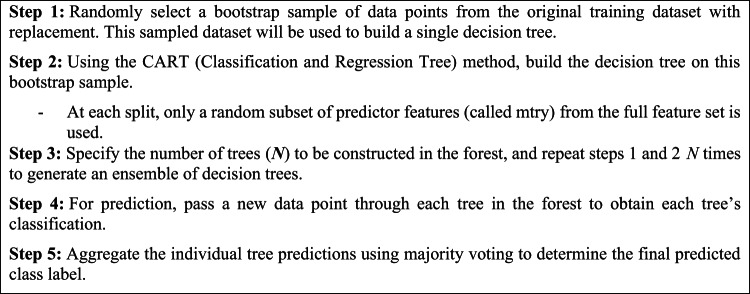
.

### Adaptive boosting (AdaBoost)

AdaBoost, the first boosting implementation, is a valuable boosting algorithm that uses shallow decision trees as base classifiers. It iteratively reweights training data to focus on previously misclassified samples, improving the model without compromising earlier classifiers. This method creates accurate, flexible models in a short amount of time^40^.

The original AdaBoost algorithm was initially designed for binary classification problems, where the base classifiers predict the probability of a target class. In this method, the weight of each instance is adjusted proportionally to its probability of being correctly predicted and indirectly proportional to the error of the classifier. In addition, the decision of each classifier on the final prediction of a new example is also weighted by its accuracy during the training phase. Along with this method, a multi-class variant, called AdaBoost.M1, was proposed in^41^. Algorithm 1 shows the pseudo-code of Adaboost.M1. In this version, only the weights of the correctly predicted instances are decreased, as shown in Line 8. This decrease is still related to the error made by the base learner (Line 5). The predictions of each classifier are still weighted by their accuracy, as seen in Line 6 and Line 15.

**Figure:**
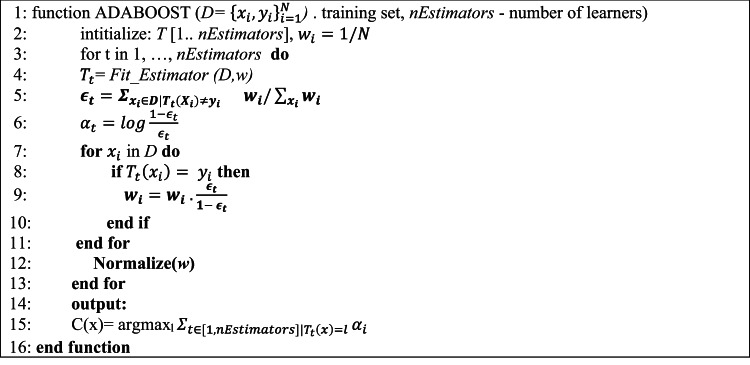
AdaBoost.M1 algorithm

### Gradient boosting machine (GBM)

Gradient Boosting Machine (GBM) is a powerful ensemble learning technique that builds models in a stage-wise and additive manner. Each stage of the algorithm fits a new base learner to the residual errors of the combined ensemble learned so far. Conceptually, the process can be interpreted as performing steepest descent optimization with respect to a specified differentiable loss function.

One of the key strengths of GBM is its flexibility, as it can be applied to both regression and classification tasks with any loss function that is differentiable. In classification problems, GBM typically fits an additive logistic regression model, where the loss function is often the negative binomial log-likelihood for binary classification or the multinomial deviance for multi-class classification^37^.

The general form of the GBM additive model is:

$$\:{\boldsymbol{F}}_{\boldsymbol{m}}\left(\boldsymbol{x}\right)=\sum\:_{m=1}^{M}{\boldsymbol{\gamma\:}}_{\boldsymbol{m}}{\boldsymbol{h}}_{\boldsymbol{m}}\left(\boldsymbol{x}\right)$$

Where $\:{h}_{m}\left(x\right)$ are the *m*_*th*_ weak learners (i.e., decision trees) that their contribution is controlled by a learning rate ($\:{\gamma\:}_{m})$. The model is built iteratively in a forward stage-wise fashion:

$$\:{\boldsymbol{F}}_{\boldsymbol{m}}\left(\boldsymbol{x}\right)={\boldsymbol{F}}_{\boldsymbol{m}-1}\left(\boldsymbol{x}\right)+{\boldsymbol{\gamma\:}}_{\boldsymbol{m}}{\boldsymbol{h}}_{\boldsymbol{m}}\left(\boldsymbol{x}\right)$$

The weak learner $\:{h}_{m}\left(x\right)$ is chosen at each iteration so that loss function *L* is minimal. To achieve this goal the model becomes:

$$\:{\boldsymbol{F}}_{\boldsymbol{m}}\left(\boldsymbol{x}\right)={\boldsymbol{F}}_{\boldsymbol{m}-1}\left(\boldsymbol{x}\right)+\underset{\boldsymbol{h}}{\mathbf{argmin}}\sum\:_{\boldsymbol{i}=1}^{\boldsymbol{n}}\boldsymbol{L}({\boldsymbol{y}}_{\boldsymbol{i}},{\boldsymbol{F}}_{\boldsymbol{m}-1}\left({\boldsymbol{x}}_{\boldsymbol{i}}\right)-{\boldsymbol{h}}_{\boldsymbol{m}}(\boldsymbol{x}\left)\right)$$

The improvement of minimization is guided by the negative gradient of the loss function with respect to the current prediction function $\:{F}_{m-1}$.

$$\:\boldsymbol{F}\left(\boldsymbol{x}\right)={\boldsymbol{F}}_{\boldsymbol{m}-1}\left(\boldsymbol{x}\right)+{\boldsymbol{\gamma\:}}_{\boldsymbol{m}}\sum\:_{\boldsymbol{i}=1}^{\boldsymbol{n}}{\nabla\:}_{\:\boldsymbol{F}}\boldsymbol{L}({\boldsymbol{y}}_{\boldsymbol{i}},{\boldsymbol{F}}_{\boldsymbol{m}-1}\left({\boldsymbol{x}}_{\boldsymbol{i}}\right))$$

The following equation is used to detect the optimal step length *γ*_*m*_ :

$$\:{\boldsymbol{\gamma\:}}_{\boldsymbol{m}}=\underset{\boldsymbol{\gamma\:}}{\mathbf{a}\mathbf{r}\mathbf{g}\mathbf{m}\mathbf{i}\mathbf{n}}\sum\:_{\boldsymbol{i}=1}^{\boldsymbol{n}}\boldsymbol{L}({\boldsymbol{y}}_{\boldsymbol{i}},{\boldsymbol{F}}_{\boldsymbol{m}-1}\left({\boldsymbol{x}}_{\boldsymbol{i}}\right)-\boldsymbol{\gamma\:}\frac{\partial\:\boldsymbol{L}({\boldsymbol{y}}_{\boldsymbol{i}},{\boldsymbol{F}}_{\boldsymbol{m}-1}\left({\boldsymbol{x}}_{\boldsymbol{i}}\right))}{\partial\:\:{\boldsymbol{F}}_{\boldsymbol{m}-1}\left({\boldsymbol{x}}_{\boldsymbol{i}}\right))})$$

This procedure is generally applicable to both regression and classification tasks; the only difference lies in the choice of the loss function^42^.

For multi-class problems, GBM approximates an additive function () for each class guided by the following loss function :

$$\:\mathcal{L}\mathcal{\:}{\left\{{\boldsymbol{y}}_{\boldsymbol{i}},{\boldsymbol{F}}_{\boldsymbol{l}}\left(\boldsymbol{x}\right)\right\}}_{1}^{\boldsymbol{L}}=\:-\:{\sum\:}_{\boldsymbol{l}=1}^{\boldsymbol{L}}\boldsymbol{l}\boldsymbol{o}\boldsymbol{g}{\:\boldsymbol{y}}_{\boldsymbol{i}}{\boldsymbol{p}}_{\boldsymbol{i}}\:\left(\boldsymbol{x}\right)$$

Where is the number of classes, takes the value 1 when sample belongs to class or 0, otherwise, and () is the probability of for the class. This probability () is estimated by the method as follows:

37 $$\:{\boldsymbol{p}}_{\boldsymbol{l}}\left(\boldsymbol{x}\right)=\:\frac{{\boldsymbol{e}}^{\boldsymbol{F}{\boldsymbol{l}}_{\left(\boldsymbol{x}\right)}}}{{\sum\:}_{\boldsymbol{j}=1}^{\boldsymbol{L}}{\boldsymbol{e}}^{\boldsymbol{F}{\boldsymbol{j}}_{\left(\boldsymbol{x}\right)}}}$$

#### Extreme gradient boosting (XGBoost)

Extreme Gradient Boosting (XGBoost) is an advanced, optimized implementation of gradient boosting algorithms, particularly designed for performance and scalability^43^. While traditional Gradient Boosting Decision Trees (GBDT) rely on the first-order derivative (gradient) of the loss function, XGBoost leverages both the first and second-order derivatives by performing a second-order Taylor expansion of the loss function. This allows for more precise and efficient model optimization.

Each tree in XGBoost is trained on residuals from the previous iteration, with the goal of progressively minimizing the overall prediction error. Unlike classical GBDT, which builds trees sequentially, XGBoost constructs trees in parallel, similar to the Random Forest approach, enabling significant computational efficiency. The way that the XGboost works is as follows: for a given data set with *n* examples and *m* features, defined as *D* = {(*x*_*i*_, *y*_*i*_)} where |D| = n, *x*_*i*_ ∈$\:{\mathbb{R}}^{m}$, *y*_*i*_ ∈$\:\mathbb{R}$, the tree ensemble model predicts the output by using the sum of *K* additive functions:

$$\:{\widehat{\boldsymbol{y}}}_{\boldsymbol{i}}=\boldsymbol{\Phi\:}\left({\boldsymbol{x}}_{\boldsymbol{i}}\right)=\sum\:_{\boldsymbol{K}=1}^{\boldsymbol{K}}{\boldsymbol{f}}_{\boldsymbol{k}}\left({\boldsymbol{x}}_{\boldsymbol{i}}\right),\:\:{\boldsymbol{f}}_{\boldsymbol{k}}\in\:\mathcal{\:}\mathcal{F}\mathcal{\:}$$

Here, $\:\mathcal{F}=\left\{f\left(x\right)={w}_{q\left(x\right)}\right\},\:where;\:q:{\mathbb{R}}^{m}\to\:T$ with q representing the structure of each tree and the number of leaves is *T*, and $\:w\:\epsilon\:{\mathbb{R}}^{T}$ represents the scores on the leaves. Each $\:{f}_{k}$ denotes an independent Classification and Regression Tree (CART), and the final prediction is obtained by summing the scores from the corresponding leaves. To learn these functions, XGBoost minimizes a regularized objective function:

$$\:\mathcal{L}\left(\boldsymbol{\varnothing\:}\right)=\sum\:_{\boldsymbol{i}}\boldsymbol{l}\left({\widehat{\boldsymbol{y}}}_{\boldsymbol{i}},{\boldsymbol{y}}_{\boldsymbol{i}}\right)+\sum\:_{\boldsymbol{k}}\boldsymbol{\Omega\:}\left({\boldsymbol{f}}_{\boldsymbol{k}}\right)$$

Here, *l* is a differentiable convex loss function, and the regularization term is defined as:

$$\:\boldsymbol{\Omega\:}\left(\boldsymbol{f}\right)=\boldsymbol{\gamma\:}\boldsymbol{{\rm\:T}}+\frac{1}{2}\:\boldsymbol{\lambda\:}{\parallel\boldsymbol{w}\parallel}^{2}$$

This regularization helps control model complexity, encouraging simpler trees and reducing overfitting. At each iteration t, a new function *f*_*t*_ is added to improve the current model, and the objective becomes:

$$\:{\mathcal{L}}^{\left(\boldsymbol{t}\right)}=\sum\:_{\boldsymbol{i}=1}^{\boldsymbol{n}}\boldsymbol{l}\left({\boldsymbol{y}}_{\boldsymbol{i}},{\widehat{\boldsymbol{y}}}_{\boldsymbol{i}}^{(\boldsymbol{t}-1)}+{\boldsymbol{f}}_{\boldsymbol{t}}\left({\boldsymbol{x}}_{\boldsymbol{i}}\right)\right)+\boldsymbol{\Omega\:}\left({\boldsymbol{f}}_{\boldsymbol{t}}\right)$$

Where $\:{\widehat{y}}_{i}^{(t-1)}$ represents the prediction of $\:i$ at iteration *t-1*, and ($\:{y}_{i},$ $\:{\widehat{y}}_{i}^{(t-1)}$) is the training loss function^44^.

### Evaluation metrics

A confusion matrix was constructed to evaluate the performance of the multiclass classification algorithms. From the classification outcomes, several performance metrics were calculated, including accuracy, precision, recall, F1 score, and the area under the ROC curve (AUC). In the confusion matrix, correctly classified instances are recorded as true positives (TP) and true negatives (TN). A false positive (FP) occurs when a negative instance is incorrectly classified as positive, while a false negative (FN) occurs when a positive instance is incorrectly classified as negative. The efficiency of the classifier is evaluated and calculated using the following formulas:

$$\:\boldsymbol{P}\boldsymbol{e}\boldsymbol{r}\boldsymbol{c}\boldsymbol{i}\boldsymbol{s}\boldsymbol{i}\boldsymbol{o}\boldsymbol{n}=\:\frac{\boldsymbol{T}\boldsymbol{P}}{\boldsymbol{T}\boldsymbol{P}+\boldsymbol{F}\boldsymbol{P}}$$

$$\:\boldsymbol{R}\boldsymbol{e}\boldsymbol{c}\boldsymbol{a}\boldsymbol{l}\boldsymbol{l}=\:\frac{\boldsymbol{T}\boldsymbol{P}}{\boldsymbol{T}\boldsymbol{P}+\boldsymbol{F}\boldsymbol{N}}$$

The formula of the *F*-measure, also known as the F1 score, is defined as:

$$\:\boldsymbol{F}-\boldsymbol{m}\boldsymbol{e}\boldsymbol{a}\boldsymbol{s}\boldsymbol{u}\boldsymbol{r}\boldsymbol{e}=2\boldsymbol{*}\:\frac{\boldsymbol{p}\boldsymbol{r}\boldsymbol{e}\boldsymbol{c}\boldsymbol{i}\boldsymbol{s}\boldsymbol{i}\boldsymbol{o}\boldsymbol{n}\boldsymbol{*}\boldsymbol{r}\boldsymbol{e}\boldsymbol{c}\boldsymbol{a}\boldsymbol{l}\boldsymbol{l}}{\boldsymbol{p}\boldsymbol{r}\boldsymbol{e}\boldsymbol{c}\boldsymbol{i}\boldsymbol{s}\boldsymbol{i}\boldsymbol{o}\boldsymbol{n}+\boldsymbol{r}\boldsymbol{e}\boldsymbol{c}\boldsymbol{a}\boldsymbol{l}\boldsymbol{l}}$$

Finally, the total classification accuracy is calculated using the following formula:

$$\:\boldsymbol{A}\boldsymbol{c}\boldsymbol{c}\boldsymbol{u}\boldsymbol{r}\boldsymbol{a}\boldsymbol{c}\boldsymbol{y}=\:\frac{\boldsymbol{T}\boldsymbol{P}+\boldsymbol{T}\boldsymbol{N}}{\boldsymbol{T}\boldsymbol{P}+\boldsymbol{F}\boldsymbol{N}+\boldsymbol{T}\boldsymbol{N}+\boldsymbol{F}\boldsymbol{P}}\boldsymbol{*}100\boldsymbol{\%}$$

## Results

In this study, the effect of class imbalance on classification performance was investigated using multiclass imbalanced LSD data, along with an evaluation of the effectiveness of various resampling techniques in addressing this issue.

### Comparing the performance of the models under the default condition and after tuning

The comparison between default and tuned conditions highlights the significant impact of hyperparameter tuning on model performance. Table 4 shows that, with default settings, training accuracy ranges from 72.5% to 84.86%, with XGBoost achieving the highest accuracy (84.86%). However, test accuracies are lower due to overfitting concerns. All models show high recall for predicting healthy cases, but recall for the “dead” class is low, with RF achieving the highest recall at 0.35 on the test set. Notably, RF consistently outperformed other models, achieving 83.65% test accuracy, perfect precision for the “dead” class (1.00), and the highest AUC value (0.92). Conversely, DT and GBoost show inconsistent precision, including undefined values (NA) for the “dead” class, and GBoost struggles with both sensitivity and precision, highlighting its difficulty in handling class imbalance.

**Table 4:** Evaluation metrics of ensemble machine learning algorithms using imbalanced data under default and tuned hyperparameter settings.

Ensemble model	Stage	Class	Accuracy		Precision		Recall		F1-score		AUC	
			Default	Tuned	Default	Tuned	Default	Tuned	Default	Tuned	Default	Tuned
DT	Training	Healthy	73%	77%	0.79	0.85	0.84	0.83	0.81	0.84	0.86	0.86
		Diseased			0.75	0.74	0.58	0.69	0.65	0.71		
		Dead			NA	0.29	NA	0.78	NA	0.42		
	Test	Healthy	80%	77.4%	0.83	0.83	0.90	0.86	0.86	0.84	0.85	0.84
		Diseased			0.85	0.73	0.65	0.59	0.74	0.66		
		Dead			NA	0.22	NA	1.00	NA	0.36		
RF	Training	Healthy	84.52%	86%	**0.92**	0.90	0.87	0.90	0.89	0.90	0.95	0.96
		Diseased			**0.75**	0.81	0.88	0.87	0.81	0.87		
		Dead			**0.85**	**0.79**	**0.35**	**0.47**	0.49	0.47		
	Test	Healthy	83.65%	85.58%	**0.92**	0.93	0.85	0.87	0.89	0.90	0.92	0.92
		Diseased			**0.68**	0.71	0.89	0.89	0.77	0.79		
		Dead			**1.00**	**0.75**	**0.22**	**0.33**	0.36	0.46		
AdaBoost	Training	Healthy	78.63%	78.63%	0.84	0.83	0.88	0.89	0.86	0.86	0.90	0.97
		Diseased			0.68	0.70	0.70	0.69	0.69	0.70		
		Dead			0.77	0.73	**0.28**	**0.17**	0.33	0.27		
	Test	Healthy	74%	76%	0.82	0.82	0.83	0.86	0.83	0.84	0.87	0.93
		Diseased			0.60	0.65	0.67	0.67	0.63	0.66		
		Dead			0.67	0.75	**0.17**	**0.25**	0.27	0.38		
GBoost	Training	Healthy	72.5%	81%	0.80	0.86	0.82	0.90	0.81	0.88	0.58	0.96
		Diseased			0.58	0.74	0.66	0.73	0.62	0.73		
		Dead			0.000	0.65	**0000**	**0.31**	NA	0.42		
	Test	Healthy	69.71%	77%	0.80	0.83	0.80	0.85	0.80	0.84	0.54	0.93
		Diseased			0.53	0.66	0.63	0.69	0.58	0.67		
		Dead			NA	0.80	**0.000**	**0.33**	NA	0.47		
XGBoost	Training	Healthy	84.86%	87%	0.86	0.91	0.94	0.92	0.90	0.91	0.93	0.97
		Diseased			0.83	0.81	0.75	0.84	0.79	0.83		
		Dead			0.76	0.82	**0.54**	**0.58**	0.63	0.68		
	Test	Healthy	74.41%	84.60%	0.81	0.87	0.84	0.94	0.83	0.90	0.87	0.93
		Diseased			0.67	0.88	0.61	0.74	0.64	0.80		
		Dead			0.31	0.53	**0.35**	**0.62**	0.33	0.57		

“NA”: Metric could not be calculated due to zero instances of this class.

After tuning the models with optimized hyperparameters, RF achieved the highest overall test accuracy (85.58%), while XGBoost demonstrated marked improvement across multiple metrics, particularly in predicting the minority “Dead” class. Although all models maintained high sensitivity for healthy cases after tuning, led by XGBoost at 0.94, the sensitivity for diseased cases varied from 0.59 (DT) to 0.89 (RF). Predicting the dead class remained challenging; AdaBoost performed the worst (test sensitivity of 0.25), while XGBoost improved to 0.62, outperforming RF (0.33) and other models. Both AdaBoost and XGBoost recorded the highest average AUCs (0.93), while DT exhibited the lowest AUC values. Overall, XGBoost stood out as the top-performing model, achieving high accuracy, recall, and F1-scores across classes. Nevertheless, RF proved to be the most balanced model, delivering the highest test accuracy and precision while maintaining robust performance across all metrics. Despite these improvements, detecting minority classes remains challenging, emphasizing the ongoing need for effective data balancing strategies to develop clinically reliable predictive models.

### Effect of resampling methods with and without tuning

**Fig. 1:**
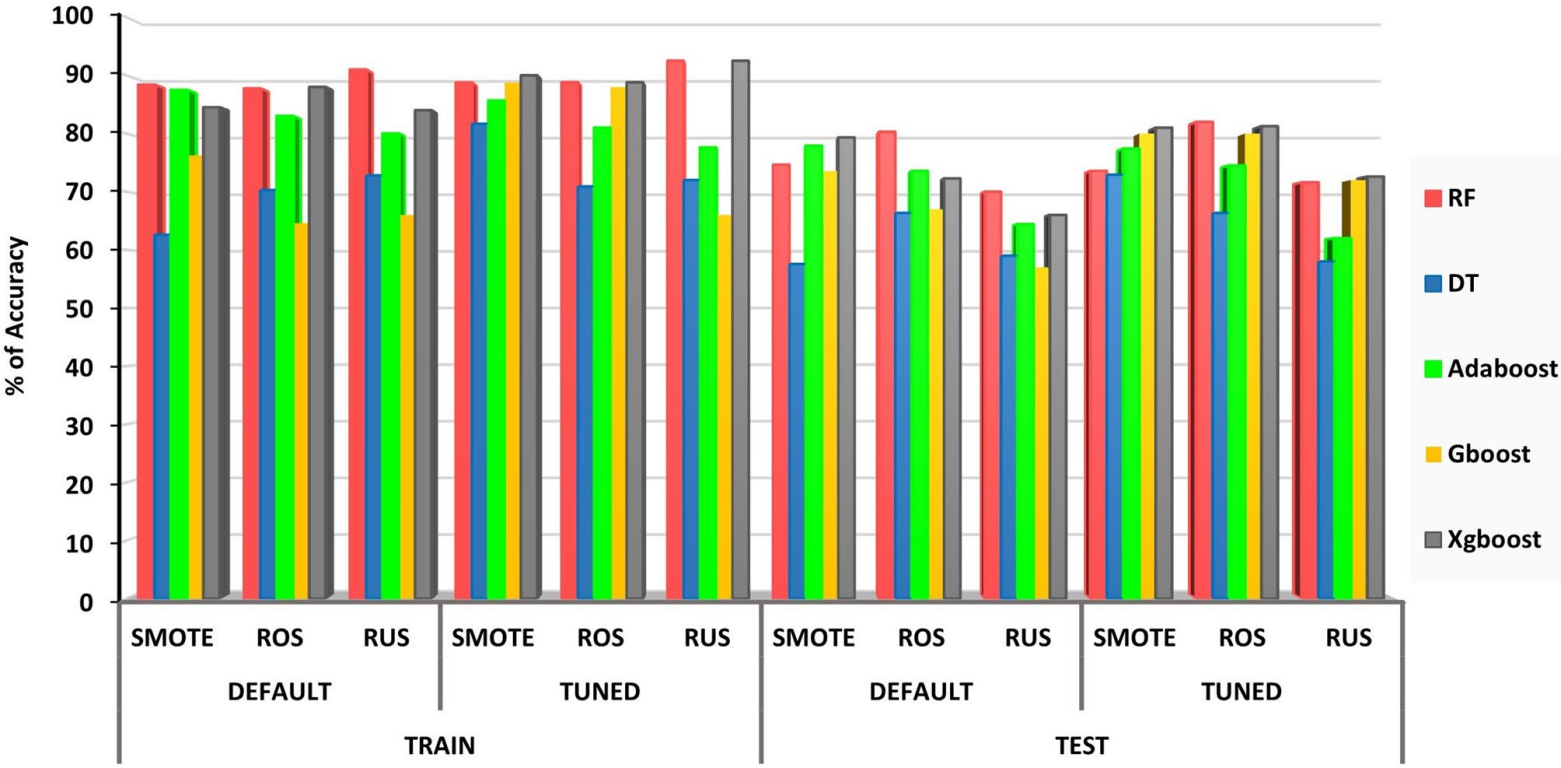
Comparison of the three resampling methods into different conditions with and without tuning of data.

We evaluated three resampling techniques: Random Over-Sampling (ROS), Random Under-Sampling (RUS), and Synthetic Minority Over-sampling Technique (SMOTE) to address class imbalance in the dataset. Among them, ROS emerged as the most effective, consistently improving both training and test performance across models. Random Forest (RF) showed the most stable and balanced behavior regardless of the resampling strategy. In contrast, XGBoost, despite achieving the highest training accuracy, was prone to overfitting under RUS. SMOTE, although theoretically robust, introduced synthetic noise that led to inconsistent generalization (Fig. 1). Given its superior performance, ROS was used to develop the final models. As shown in Table 5, RF achieved the best overall results, with training and test accuracies of 87.75% and 80.29%, respectively, indicating strong generalizability. While XGBoost attained the highest training accuracy (88%), its test accuracy declined sharply to 72.28%, reinforcing the presence of overfitting. Decision Tree yielded the lowest performance, with training and test accuracies of 70.29% and 66.35%, respectively, reflecting limited generalization and sensitivity to noise. These results highlight that while data balancing can enhance performance, model responses vary. RF generalized well, but models like XGBoost and DT remained susceptible to overfitting or underfitting, underscoring the need for model-specific strategies such as hyperparameter tuning beyond resampling alone.

**Table 5:** Evaluation metrics of ensemble machine learning algorithms using ROS data under default and tuned hyperparameter settings.

Ensemble model	Stage	Class	Accuracy		Precision		Recall		F1-score		AUC	
			ROS	ROS + Tuning	ROS	ROS + Tuning	ROS	ROS + Tuning	ROS	ROS + Tuning	ROS	ROS + Tuning
DT	Training	**Healthy**	70.29%	70.29%	0.64	0.64	0.87	0.87	0.74	0.74	0.83	0.83
		**Diseased**			0.64	0.64	0.63	0.63	0.64	0.64		
		**Dead**			0.82	0.82	**0.66**	**0.66**	0.74	0.74		
	**Test**	**Healthy**	66.35%	66.35%	0.66	0.66	0.95	0.95	0.78	0.78	0.82	0.82
		**Diseased**			0.70	0.70	0.52	0.52	0.60	0.60		
		**Dead**			0.44	0.44	**0.12**	**0.12**	0.19	0.19		
RF	Training	**Healthy**	87.75%	88.8%	0.94	0.95	0.82	0.84	0.87	0.89	0.95	0.95
		**Diseased**			0.86	0.84	0.81	0.85	0.84	0.85		
		**Dead**			0.85	0.88	**1.00**	**0.97**	0.92	0.92		
	**Test**	**Healthy**	80.29%	82%	0.95	0.96	0.80	0.80	0.87	0.87	0.90	0.93
		**Diseased**			0.64	0.65	0.79	0.86	0.70	0.74		
		**Dead**			0.44	0.57	**0.89**	**0.89**	**0.59**	0.70		
AdaBoost	Training	**Healthy**	83%	81%	0.92	0.81	0.76	0.79	0.83	0.80	0.90	0.97
		**Diseased**			0.77	0.80	0.78	0.71	0.78	0.75		
		**Dead**			0.83	0.82	**0.96**	**0.93**	0.89	0.87		
	**Test**	**Healthy**	73.56%	74.5%	0.92	0.91	0.73	0.78	0.81	0.84	0.84	0.92
		**Diseased**			0.55	0.57	0.75	0.68	0.63	0.62		
		**Dead**			0.39	0.28	**0.78**	**0.56**	0.52	0.37		
GBoost	Training	**Healthy**	64.6%	88%	0.82	0.92	0.67	0.84	0.74	0.88	0.96	0.97
		**Diseased**			0.55	0.88	0.66	0.80	0.60	0.84		
		**Dead**			0.61	0.85	**0.61**	**1.00**	0.61	0.92		
	**Test**	**Healthy**	67%	82%	0.89	0.93	0.71	0.83	0.79	0.87	0.92	0.97
		**Diseased**			0.57	0.65	0.64	0.73	0.61	0.69		
		**Dead**			0.19	0.44	**0.33**	**0.89**	0.15	0.59		
XGBoost	Training	**Healthy**	88%	88.8%	0.93	0.94	0.82	0.84	0.87	0.89	0.97	0.97
		**Diseased**			0.85	0.85	0.84	0.85	0.84	0.85		
		**Dead**			0.87	0.88	**0.97**	**0.97**	0.92	0.92		
	**Test**	**Healthy**	72.28%	81.25%	0.88	0.93	0.77	0.82	0.82	0.87	0.93	0.93
		**Diseased**			0.63	0.65	0.64	0.79	0.64	0.71		
		**Dead**			0.26	0.57	**0.65**	**0.89**	0.38	0.70		

When combined with resampling, hyperparameter tuning substantially improved model performance. Notably, RF and XGBoost achieved the highest test accuracies (82% and 81.25%) while maintaining strong training accuracy (88.8%). Although AdaBoost and GBoost showed moderate gains post-tuning, they still lagged behind, suggesting limitations in adapting to the dataset’s complexity. All models performed well in identifying healthy cases (recall > 0.78); however, only RF and XGBoost achieved high recall across all classes. RF notably reached a recall of 0.89 for the “Dead” class, emphasizing its superior capacity to detect minority outcomes. Both models also exhibited high precision (≥ 0.57) and strong AUCs (0.95–0.98 training; 0.93 test), reflecting robust class discrimination. In contrast, DT and AdaBoost struggled with the “Dead” class, showing low sensitivities (0.12 and 0.56, respectively) and reduced generalization. Poor precision in DT (0.44), GBoost (0.44), and AdaBoost (0.28), along with low F1-scores for DT (0.19) and AdaBoost (0.37), further indicate their difficulty in balancing precision and recall even after ROS. These findings suggest that although resampling and tuning enhance overall performance, certain models particularly DT, AdaBoost, and GBoost, may still require further optimization through alternative resampling techniques or hybrid strategies to more effectively address severe class imbalance.

### Significant impact of hyperparameter tuning in balanced and imbalanced scenarios

The effect of hyperparameter tuning on model performance was assessed using paired t-tests comparing accuracy and AUC before and after tuning across imbalanced and balanced datasets.

For the imbalanced dataset, accuracy significantly improved for DT (*p* = 0.003), RF (*p* = 0.018), GBoost (*p* < 0.001), and XGBoost (*p* < 0.001), while AdaBoost showed no significant change (*p* = 0.137). AUC improvements were significant for AdaBoost (*p* < 0.001), GBoost (*p* < 0.001), and XGBoost (*p* < 0.001), whereas DT (*p* = 0.186) and RF (*p* = 0.585) did not exhibit significant changes. In the balanced dataset, tuning significantly enhanced accuracy for GBoost (*p* < 0.001) and XGBoost (*p* < 0.001), but not for DT (*p* = 0.160), RF (*p* = 0.074), or AdaBoost (*p* = 0.307). Regarding AUC, significant gains were found for RF (*p* = 0.005) and AdaBoost (*p* < 0.001), while DT (*p* = 0.322) and XGBoost (*p* = 1.000) remained statistically unchanged.

These findings underscore the importance of hyperparameter tuning in enhancing ensemble model performance, particularly for boosting algorithms, while also demonstrating the inherent robustness of Random Forest, which performed strongly under default settings and achieved further improvements in both accuracy and AUC after tuning across balanced and imbalanced datasets.

#### Computational complexity of the implemented models

The computational complexity of the algorithms was influenced by the experimental platform used in our study, which consisted of Windows 10 × 64-bit operating system, 4 GB of RAM, an Intel^®^ Core™ i5-7200U CPU @ 2.50 GHz, and the R software version 4.4.1 (2024-06-14 ucrt). The system’s memory limitations and CPU processing speed were key factors in the observed computational demands. Hyperparameter tuning, combined with 10-fold cross-validation repeated five times, introduced significant computational overhead.

Distinct variations in computational cost were observed across models. Decision Trees demonstrated the lowest demands, with training times ranging from 14 to 26 min and moderate memory usage (~ 281 MB). Random Forest (RF) required slightly more resources, with training times of 23–28 min and memory usage reaching 351 MB when applied to balanced data. In contrast, boosting algorithms exhibited significantly higher computational costs. XGBoost required over 3 h to train on imbalanced data and an additional hour for balanced data, with a peak memory usage of 735 MB. GBoost required approximately 1.15 h, while AdaBoost and its balanced counterpart took about 1 h and 1.5 h, respectively, with memory usage around 300 MB. These results indicate that simpler tree-based models demand fewer computational resources, whereas boosting algorithms, although often yielding superior performance, require substantially more time and memory. Furthermore, dataset balancing notably increased computational costs for boosting models, particularly for XGBoost. The extensive hyperparameter search during cross-validation further amplified training time across all models.

### Feature importance analysis using the random forest ensemble model

**Fig. 2:**
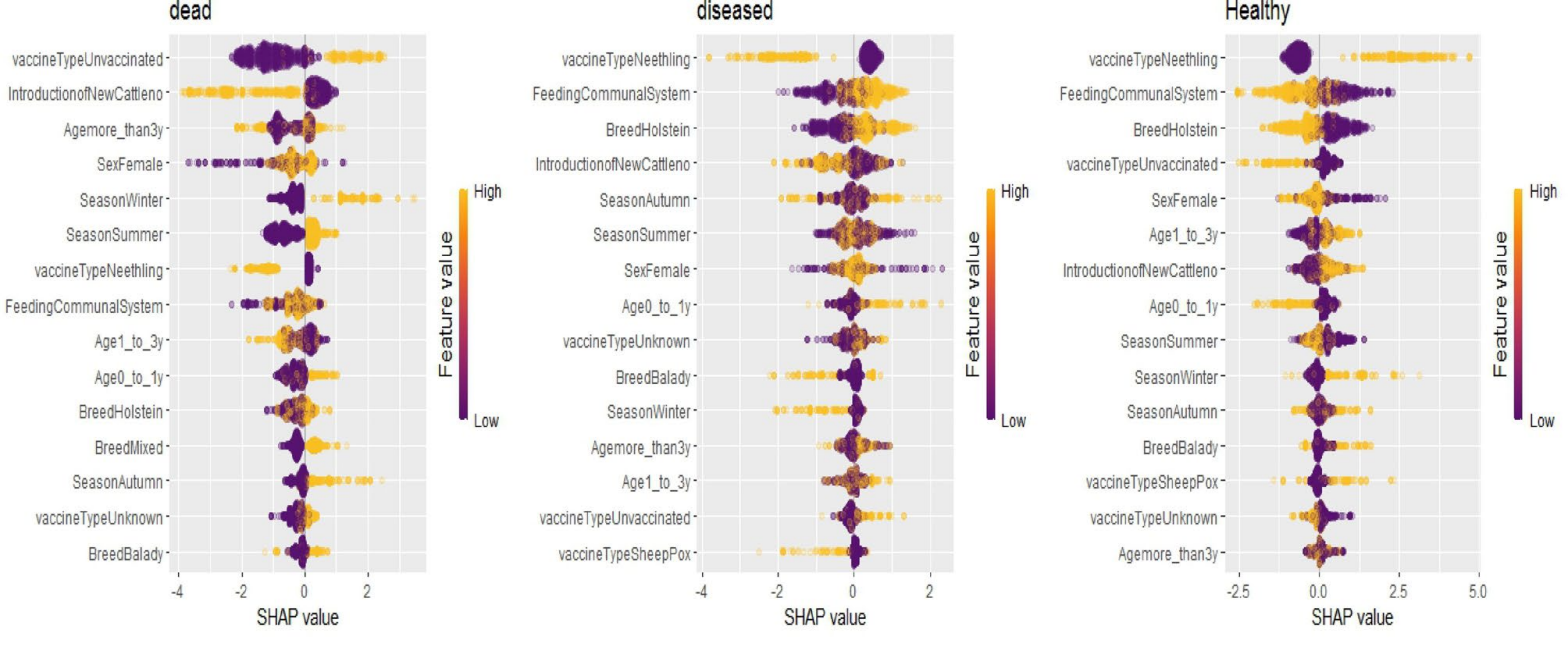
SHAP values addressing the impact of features. Each point represents a sample. The higher the SHAP value the higher the risk of LSD, and vice versa.

We conducted SHAP (SHapley Additive Explanations) analysis on the best-performing ensemble model, Random Forest. The resulting summary plot (Fig. 2) provides valuable insights into the relative importance and directional influence of the features used. By evaluating the magnitude and direction of each variable’s contribution, we gain a deeper understanding of the mechanisms that enhance model performance. Notably, the Neethling vaccine type showed the strongest positive association with the healthy class, whereas the communal feeding system was closely linked to disease presence. Seasonal patterns also emerged, with winter associated with higher mortality risks compared to summer and autumn. Age was a critical determinant, as animals under one year of age were more susceptible to infection and mortality. While breed and age contributed to the model’s predictions, their influence was less significant than that of vaccination and feeding practices. These detailed findings uncover key risk factors shaping LSD outcomes and offer a data-driven basis for designing targeted, risk-based control strategies. A comprehensive summary of these contributions and the novelty of our findings is presented in Table 6.

**Table 6:** Key contribution and novelty of our approach against the used methods.

Scenario	Approach	Performance	Key Findings	Novelty
Baseline (Default)	Ensemble models (RF, XGB, AdaBoost, etc.) without resampling or tuning.	Poor performance for minority classes; moderate overall due to high bias toward the majority class.	DT performed poorly; RF and XGBoost showed slightly better robustness.	Establishes a baseline for assessing improvements.
Improved Tuning Only	Ensemble models with hyperparameter tuning only.	Moderate improvement, especially in DT and GBoost.	Tuning improved the performance of weak learners (DT, Ada, GBoost).	Highlights the role of tuning in mitigating class imbalance effects.
Resampling Only	Resampling using ROS, RUS, SMOTE with default model parameters.	ROS > SMOTE > RUS in improving minority class recall.	Oversampling improved recall; RUS reduced overall accuracy.	Validates ROS as a simple and effective resampling method.
Combined (Resampling + Tuning)	Ensemble models + resampling + tuning.	Achieved the highest overall performance across all metrics.	RF-ROS with tuning gives the best results.	First study to evaluate and compare both resampling and tuning for multiclass LSD prediction.
Model Rankings	Across all combinations	RF > XGB > Ada > GBoost > DT.	RF dominant in precision and F1, even with imbalance.	Provides empirical evidence of model robustness.
SHAP Interpretation	Applied to the best-performing model ((RF + ROS + tuning)).	Identified vaccination status, grazing system, and season as top predictors.	Neethling vaccine linked to healthy cases.	Enhances model transparency and ML interpretability using SHAP.

## Discussion

Lumpy Skin Disease presents a major threat to livestock health and food security. Despite advancements in disease management, accurate prediction of LSD outbreaks remains a challenge. Ensemble learning techniques, such as bagging and boosting, offer promising solutions for improving predictive performance. However, limited research has systematically compared the performance of bagging and boosting models for LSD classification, particularly in the context of highly imbalanced, multiclass data. To the best of the author’s knowledge, no prior study has employed ensemble ML algorithms to forecast the risk of LSD using multiclass imbalanced data and evaluate the performance using different resampling approaches. This study addresses this gap by evaluating and comparing the predictive capabilities of bagging and boosting methods, investigating the effects of hyperparameter tuning, and assessing the effects of three resampling techniques: SMOTE, Random Oversampling (ROS), and Random Undersampling (RUS).

The data exhibited a significant class imbalance, a common challenge in machine learning. Some ensemble learning algorithms. particularly Random Forest (RF), XGBoost, and AdaBoost, performed well under default imbalanced conditions. These results are consistent with previous research showing that conventional ML algorithms often underperform on imbalanced datasets, while ensemble approaches tend to offer improved performance^45^. Similarly, Zhu et al.^46^ demonstrated that ensemble algorithms enhance predictive accuracy in medical datasets. In contrast, other algorithms were more adversely affected, showing reduced predictive accuracy and generalization when trained on imbalanced data. Notably, the Decision Tree model performed poorly, which aligns with findings by Mienye and Sun^45^, who observed that DTs perform adequately on balanced data but deteriorate under class imbalance. This performance drop has been further attributed by Silaghi and Mathew^47^ to the DT’s tendency to overfit the majority class by favoring splits that maximize information gain while neglecting minority classes.

To mitigate the detrimental effects of class imbalance, three resampling techniques: Random Oversampling, Random Undersampling, and SMOTE, were evaluated. Our results indicated that ROS consistently outperformed the other methods in terms of model accuracy. This finding aligns with previous research by Kamalov et al.^48^, who highlighted the effectiveness and computational efficiency of ROS compared to more complex techniques like SMOTE. This reinforces the notion that simpler methods can sometimes yield better results than more sophisticated approaches. In contrast, SMOTE was found to enhance the accuracy of imbalanced LSD data in a study by Venkata Pratyusha Kumari^49^. Similarly, Kim and Hwang^50^ reported that ROS and SMOTE outperformed other resampling techniques, while undersampling often led to decreased performance. Overall, oversampling appeared generally more effective than undersampling for improving classification outcomes. However, as discussed by Kovács^51^, the effectiveness of resampling strategies can vary depending on the degree of class imbalance and the specific method used. Cieslak et al.^52^ likewise emphasized that oversampling tends to outperform undersampling in scenarios involving severe imbalance.

Moreover, fine-tuning significantly improved model performance, particularly for minority classes. The most substantial gains were observed in DT, AdaBoost, and GBoost models, which initially exhibited poor performance under imbalanced conditions. Hyperparameter optimization proved essential, though its impact varied across algorithms. These findings are consistent with those of Probst et al.^53^, who reported that Gradient Boosting Machines, unlike Random Forests, exhibit considerable variability in performance depending on hyperparameter configurations, necessitating more strategic tuning. Similarly, a previous study^54^ emphasized that hyperparameter tuning effectively mitigates overfitting and enhances deep learning model performance. In contrast, Carreira-Perpiñán and Zharmagambetov^55^ noted that although RF, AdaBoost, and GBoost are generally considered robust to hyperparameter selection, some level of tuning is often necessary to achieve optimal performance, depending on dataset-specific characteristics.

Building on the results of class imbalance and tuning effects, an overall evaluation of the five ensemble algorithms revealed that RF consistently achieved the highest performance across all scenarios, whether on imbalanced or balanced datasets, and under both tuned and default settings. This demonstrates its robustness and effectiveness in addressing data-related challenges. RF particularly excelled in precision and F1 score, even within the minority class. These findings align with those of Mirzaeian et al.^56^, who reported that RF outperformed other ensemble models such as XGBoost, GBoost, and AdaBoost. Additionally, RF’s strong performance in handling imbalanced data is consistent with the previous study^38^. Among the boosting algorithms, XGBoost ranked second, reinforcing its status as a strong alternative, particularly for datasets with uneven class distributions, as noted previously by Fitriyani et al.^57^. Moreover, we observed notable improvements in accuracy, precision, and recall for both RF and XGBoost after hyperparameter tuning and data balancing, further supporting the results of a previous research^45^. Collectively, these results highlight RF and XGBoost as the most robust and high-performing models across various conditions, consistent with the conclusions drawn by Gurcan and Soylu^58^. Overall, these findings underscore the critical importance of carefully selecting both the ensemble model, tuning and resampling strategies when addressing classification tasks involving imbalanced data.

While tuning plays a vital role in optimizing model performance by ensuring the most effective parameter settings, it often entails high computational costs. This is particularly evident in boosting algorithms like XGBoost, which are more resource-intensive compared to bagging methods like RF. Computational complexity is influenced by several factors, including dataset size and structure, algorithm type, number of iterations, and hardware limitations. These observations are consistent with the findings of a previous study^59^, which emphasized that selecting optimal hyperparameters for both bagging and boosting techniques is a challenging and time-consuming process, yet crucial for enhancing classification performance. Moreover, our findings underscore that computational complexity can impact model performance, aligning with the conclusions of Ziolkowski^60^. Prior studies have also highlighted that both computational complexity and model performance can be improved through the use of resampling techniques and feature selection. For example, Khan et al.^61^ used the NearMiss method to address class imbalance, improving both reliability and computational efficiency by reducing dataset size and minimizing overfitting risks. Similarly, previous research^62^ demonstrated improved accuracy and reduced computational cost through optimal feature selection, which involved eliminating redundant or noisy variables. These insights underscore the importance of maintaining a balance between accuracy and computational efficiency.

To enhance the real-world interpretability of our ensemble learning models and to gain deeper insights into the factors contributing to LSD risk, we employed SHAP value analysis, as recommended by Gurcan and Soylu^58^. The results identified vaccination status as the most influential predictor. Animals vaccinated with the Neethling vaccine were more likely to be classified as healthy, whereas unvaccinated animals were more frequently classified as dead. This finding reflects the strong protective effect of the Neethling LSDV strain and its association with improved health outcomes, consistent with previous research^63^. In contrast, the Sheeppox vaccine demonstrated lower efficacy in reducing LSD morbidity. This may be attributed to the higher viral doses typically used in heterologous Sheeppox virus vaccines, which, although considered safe, are less effective in cattle than homologous vaccines, as noted in earlier studies^64,65^. Other key risk factors identified include grazing systems, the introduction of new animals, season, breed, and age. Communal grazing and new animal introductions significantly increased LSD risk, consistent with Selim et al.^3^. Seasonally, LSD prevalence peaked in autumn, followed by summer, aligning with previous findings^1^, that attribute this trend to warm, humid climates favorable for vector activity. However, other researchers^66^ reported a higher prevalence in winter. In our study, mortality was notably higher during winter, potentially due to stress-related factors and management challenges. This is supported by EFSA^67^, which suggested that winter LSD cases may result from vector-independent transmission routes and delays in outbreak reporting. Regarding demographic factors, sex was not a significant risk predictor, consistent with Selim et al.^3^. Age showed mixed associations; while young calves (< 1 year) were highly susceptible^68,69^, some studies indicated a higher risk in older cattle^3,66^. Conversely, other authors indicated that neither sex nor age was significantly related to LSD risk prediction^70,71^.

Based on the insights derived from the SHAP analysis, several practical disease management strategies can be proposed for more effective LSD control. The identification of vaccination status as the most influential risk factor underscores the importance of prioritizing effective vaccination campaigns, particularly with Neethling-based vaccines, which have demonstrated superior protection. In contrast, the limited effectiveness of the Sheeppox vaccine highlights the need to adopt more efficacious, homologous alternatives. Focused immunization efforts should target unvaccinated animals and young calves in high-risk areas, as informed by risk modeling. The association between communal grazing and increased LSD risk underscores the importance of promoting controlled grazing systems and raising farmer awareness. Additionally, strict quarantine measures, including disease testing and sourcing from reputable suppliers, are essential to mitigate risks linked to the introduction of new animals. Given the seasonal rise in LSD cases during autumn and summer, enhanced surveillance and vector control during these periods is warranted. Overall, these findings demonstrate how machine learning outputs can be translated into actionable, field-level recommendations, reinforcing the value of explainable AI in veterinary disease management.

### Limitations and future work

While this study demonstrates promising results in predicting LSD outcomes using ensemble ML techniques, several limitations should be acknowledged to provide a balanced perspective. The dataset comprising 1,041 samples, while informative, may limit generalizability, especially across larger or more diverse populations. Real-world variability, including environmental, management, and breed differences, was not fully captured, suggesting the need for future studies with more extensive datasets to improve robustness and applicability. The study focused on SMOTE, ROS, and RUS for resampling, but advanced techniques like Tomek Links, NearMiss, or hybrid strategies could further enhance performance in addressing class imbalance. Moreover, the current evaluation was limited to five ensemble algorithms; incorporating more diverse modeling approaches, including stacking ensembles or deep learning architectures, could offer a more comprehensive understanding of model behavior across different scenarios. Hyperparameter tuning, conducted via grid search, was computationally intensive. This, along with algorithmic design and hardware limitations, affected scalability. Future work should explore more efficient optimization methods, such as Bayesian Optimization, to reduce computational cost and enhance model scalability across larger datasets or resource-constrained environments. Another limitation is the classification of “Dead” cases, which may be subject to misclassification bias due to field diagnosis constraints. Moreover, the current framework focused solely on clinical outcomes, excluding broader economic impacts of LSD, such as reductions in milk yield, fertility, and culling rates. Future studies should aim to integrate these factors to provide a more holistic understanding of LSD’s impact. Moving forward, future work should also expand the dataset to include additional risk factors, geographic diversity, and post-resampling data cleaning techniques. Addressing these limitations will improve model precision and robustness, facilitating more informed, data-driven decisions in livestock health management.

## Conclusion

This study developed and evaluated ensemble machine learning models for predicting LSD in livestock. The findings demonstrate that ensemble algorithms, particularly Random Forest and XGBoost, can effectively predict LSD occurrence, even in the presence of class imbalance. Model performance was significantly enhanced through hyperparameter tuning and 10-fold cross-validation. The study highlights that tuning must be tailored to the algorithm and data characteristics. Boosting methods, known for their sensitivity to hyperparameters, showed the greatest gains, indicating their dependency on careful parameter optimization. Meanwhile, bagging methods like RF exhibited more stable performance but still benefited from tuning in specific contexts.

Among resampling techniques, SMOTE and ROS outperformed RUS in managing class imbalance, contributing to more reliable model outcomes. The analysis identified key risk factors for LSD, including vaccination status (with Neethling vaccine showing higher effectiveness), communal grazing, recent animal introductions, seasonal patterns (peaking in autumn and summer), breed susceptibility, and younger age groups. Notably, while vector-borne transmission remains central, vector-independent transmission especially during winter, also plays a role. By analyzing various risk factors, these models can assist farmers and decision-makers in implementing targeted prevention and control strategies. The models demonstrate significant potential to improve the accuracy of LSD predictions.

## Data Availability

The datasets generated and/or analyzed during the current study are available from the corresponding author upon reasonable request.
