# Correction: Comparative performance of bagging and boosting ensemble models for predicting lumpy skin disease with multiclass-imbalanced data

**DOI:** 10.1038/s41598-026-36175-0

**Published:** 2026-01-15

**Authors:** Hagar F. Gouda, Fatma D. M. Abdallah

**Affiliations:** Animal Wealth Development Department (Biostatistics subdivision), Faculty of Veterinary Medicine, Zagazig University, Zagazig, 44511 Sharkia Egypt

Correction to: *Scientific Reports* 10.1038/s41598-025-23846-7, published online 10 November 2025

In the original version of this Article, Figures 1 did not display correctly. The original Figure 1 and the accompanying legend appear below.

**Fig. 1:**
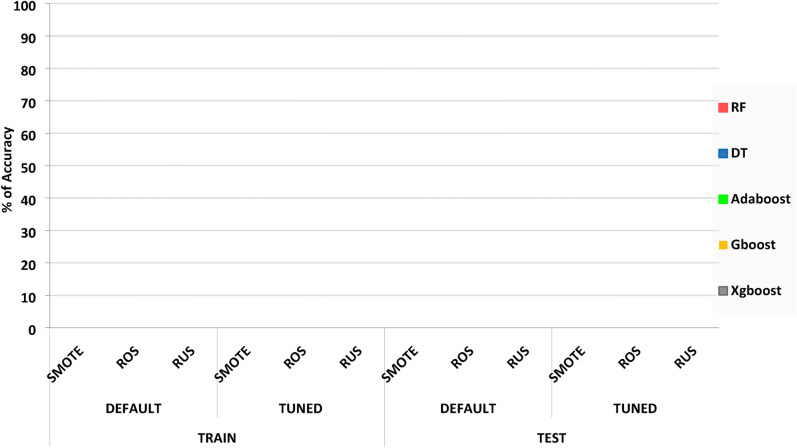
Comparison of the three resampling methods into different conditions with and without tuning of data.

The original Article has been corrected.

